# Myogenic differentiation triggers PML nuclear body loss and DAXX relocalization to chromocentres

**DOI:** 10.1038/cddis.2017.151

**Published:** 2017-03-30

**Authors:** Jayme Salsman, Lindsy M Rapkin, Nandini N Margam, Roy Duncan, David P Bazett-Jones, Graham Dellaire

**Affiliations:** 1 Department of Pathology, Dalhousie University, Halifax, NS, Canada, B3H 4R2; 2 Genetics & Genome Biology Program, The Hospital for Sick Children, Toronto, ON, Canada, M5G 1X8; 3 Department of Microbiology & Immunology, Dalhousie University, Halifax, NS, Canada, B3H 4R2; 4 Department of Biochemistry & Molecular Biology, Dalhousie University, Halifax, NS, Canada, B3H 4R2

## Abstract

The promyelocytic leukemia protein (PML) is expressed in most normal human tissues and forms nuclear bodies (NBs) that have roles in gene regulation and cellular processes such as DNA repair, cell cycle control, and cell fate decisions. Using murine C2C12 myoblasts, we demonstrate that activation of skeletal muscle differentiation results in loss of PML and PML NBs prior to myotube fusion. Myotube formation was associated with marked chromatin reorganization and the relocalization of DAXX from PML NBs to chromocentres. MyoD expression was sufficient to cause PML NB loss, and silencing of PML induced DAXX relocalization. Fusion of C2C12 cells using the reptilian reovirus p14 fusogenic protein failed to disrupt PML NBs yet still promoted DAXX redistribution and loss; whereas ectopic expression of PML in differentiated cells only partially restored PML NB formation and DAXX localization at NBs. Finally, we determined that the C-terminal SUMO-interacting motif of DAXX is required for its colocalization with ATRX in heterochromatin domains during myotube formation. These data support a model in which activation of myogenic differentiation results in PML NB loss, chromatin reorganization and DAXX relocalization, and provides a paradigm for understanding the consequence of PML loss in other cellular contexts, such as during cancer development and progression.

Originally identified and characterized as a tumor suppressor, the promyelocytic leukemia (PML) protein has an important role in the regulation of gene expression.^1^ The six nuclear-localized human PML protein isoforms can form multimeric structures that provide the underlying scaffold for the formation of PML nuclear bodies (PML NBs). SUMO modification of PML is important for PML NB formation and recruitment of SUMO-interacting motif (SIM)-containing PML NB components such the histone chaperone DAXX.^2, 3^ PML NBs associate with over 150 different cellular proteins,^4, 5^ either constitutively such as transcriptional repressors SP100 and DAXX,^2, 6^ or transiently in response to stress such as the DNA repair factors MRE11 and TOPBP1.^7, 8, 9^ The majority of these proteins are involved in regulating gene expression as transcription factors (e.g., STAT3, SP1, GATA2), transcription factor modifying enzymes (e.g., PIAS1, SENP1, HIPK2) or chromatin modifiers (e.g., DAXX, CBP, HDACs).^4, 5^ Although PML NBs are devoid of nucleic acids and primarily reside in the inter-chromosomal space,^10^ they make contacts with specific chromatin regions on their surface,^11^ and are associated with transcriptionally active chromatin.^12^ These data have led to the hypothesis that PML NBs act as ‘hubs' for the recruitment and modification of transcription factors and as such, PML NBs coordinate a variety of cellular responses such as DNA repair, cell cycle control, cell fate decisions (apoptosis, senescence), and interferon signaling^7, 13^

During development, the coordinated execution of well-controlled gene expression programs are required to establish specific cell and tissue types, and a role for nuclear structure in this process is only now being explored. One such process is skeletal muscle development. Through the coordinated, sequential activation of multiple transcription factors, allowing myoblasts to differentiate into multinucleated myotubes that form the structural basis of skeletal muscle.^14, 15^ For example, C2C12 myoblasts express the partially redundant transcription factors MyoD (MYOD1) and MYF5 committing them to the myoblast phenotype.^16, 17, 18^ When C2C12 myoblasts are grown in the presence of mitogens (10% FBS), the inhibitor of differentiation protein is expressed and blocks differentiation by inhibiting myogenic factors.^19^ Under conditions of mitogen deprivation, including 2% horse serum, proliferating myoblasts can withdraw permanently from the cell cycle,^20^ induce expression of myogenin, become committed to the myogenesis differentiation program, and begin to express muscle-specific genes such as myosin heavy chain.^15, 21, 22, 23^ The cells expressing muscle-specific genes align and fuse to form multinucleated syncytial myotubes. The entire process requires the temporally coordinated differential expression of hundreds of genes.^24, 25^

Although PML null mice are born with expected Mendelian frequency, the terminal differentiation capacity of their myeloid cells is impaired and PML is required for hematopoietic stem cell maintenance.^26, 27^ In addition, mammary epithelial differentiation and alveolar development is aberrant,^28^ and neural progenitor cells have impaired function in these animals.^29^ Together, these findings provide compelling evidence that PML may be important for regulating developmental processes in other organs including skeletal muscle. Using the well-characterized murine C2C12 cells for modelling myogenesis *in vitro,* we investigated both the fate and contribution of PML and PML NBs to the muscle differentiation program.

## Results

**Figure 1:**
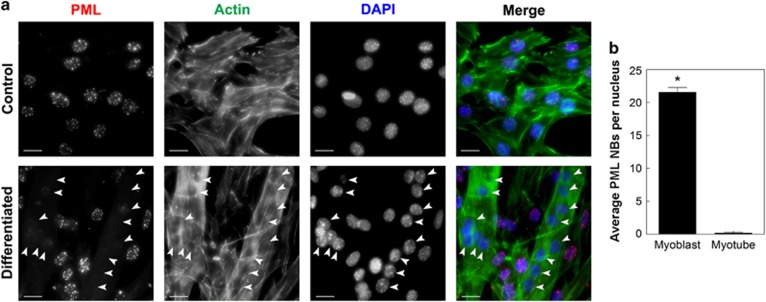
PML nuclear body loss is associated with C2C12 differentiation. (**a**) C2C12 myoblast cells were cultured in growth media (control) or differentiation media for 4 days (differentiated). Formaldehyde-fixed cells were immunostained for PML, actin was visualized with fluorophore-conjugated phalloidin and DNA was visualized with DAPI. Syncytial nuclei associated with myotubes are indicated with arrowheads. Scale bar=20 *μ*m. (**b**) Quantification of PML NBs in C2C12 myoblasts and syncytial nuclei in myotubes. Data are presented as the mean±standard error, *n*=3, *P<0.01

In order to investigate the role of PML NBs in myogenesis we used the C2C12 mouse muscle myoblast cell line, where substituting mitogen-rich FBS with 2% mitogen-poor horse serum (i.e., differentiation media; DM) can induce their differentiation into multinucleated myotubes. Undifferentiated myoblast cell nuclei (Figure 1a, control) contained a wide range of PML NBs with an average of ~22 PML NBs per cell (Figure 1b). After 4 days of horse serum treatment, many cells had differentiated and fused into elongated, multinucleated myotubes that were visualized by actin staining (Figure 1a, differentiated). Strikingly, nuclei within syncytial myotubes showed greatly reduced (1–5 PML NBs/nucleus) or complete loss of PML (Figure 1a, arrowheads, Figure 1b).

**Figure 2:**
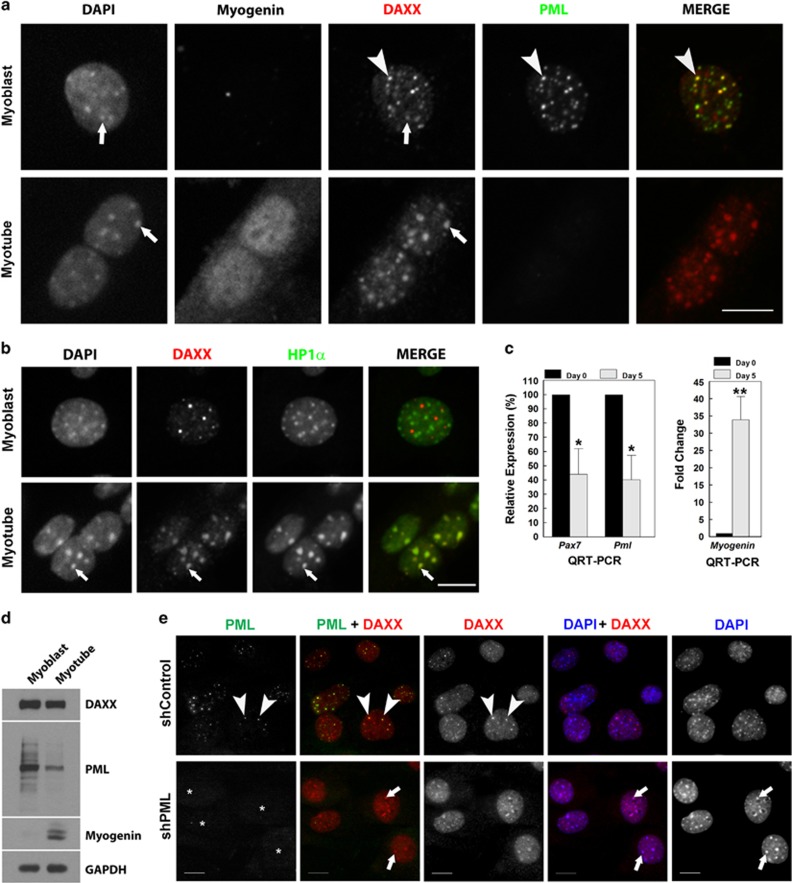
DAXX localizes to PML NBs in myoblasts and chromocentres in myotubes. (**a**) C2C12 myoblasts and myotubes were immunostained for DAXX, PML, and myogenin as indicated. DNA was visualized with DAPI. Examples of colocalization between PML and DAXX are indicated with arrowheads (>). Examples of colocalization between DNA and DAXX are indicated with arrows (**→**). (**b**) C2C12 myoblasts and myotubes were immunostained for DAXX and HP1*α*. DNA was visualized with DAPI. Examples of colocalization between DNA, HP1*α*, and DAXX are indicated with arrows. (**c**) Quantitative RT-PCR for *Pml, Pax7* (myoblast expressed), and *Myogenin* (myotube expressed) mRNA expression in C2C12 cells at day 0 (undifferentiated) and day 5 post differentiation. Data are presented as the mean±standard error,*n*=3, *P<0.05, **P<0.01. (**d**) Detection of PML and DAXX protein expression in C2C12 myoblasts and myotubes as assessed by western blot analysis. (**e**) C2C12 cells were infected with lentivirus encoding either control shRNA (shControl) or shRNA-targeting PML (shPML). Cells were immunostained for PML and DAXX as indicated. DNA was visualized with DAPI. Arrowheads (>) indicate colocalization between PML and DAXX. Arrows (**→**) indicate colocalization between DAXX and chromocentres. Asterisks indicate the location of nuclei. Scale bars=10 *μ*m

As previous studies indicate that lack of PML and PML NBs results in redistribution of constitutive PML NB components such as DAXX,^2, 30^ we next examined DAXX localization in C2C12 myoblasts and myotubes (Figure 2a). In myoblasts, DAXX was primarily associated with PML NBs (Figure 2a, arrowheads) with some DAXX association with regions of condensed chromatin as indicated by DAPI staining (Figure 2a, arrow). In myogenin-positive myotubes, PML NBs were absent and DAXX relocalized to DAPI-enriched condensed chromatin (Figure 2a, arrows). This staining pattern was similar to that of PML –/− mouse primary embryonic fibroblasts where DAXX is associated with chromocentres and colocalizes with HP1*α*.^2, 30^ As well, myogenic differentiation is associated with large-scale chromatin reorganization and chromocentre clustering,^31, 32^ which can be observed with DAPI staining of DNA (Figure 2a, arrows). We confirmed that, in myotubes, but not myoblasts, DAXX and the heterochromatin-associated protein HP1*α* colocalize at chromocentres (Figure 2b, arrows). Myogenic differentiation was also associated with reduced PML mRNA (Figure 2c) and total PML protein (Figure 2d) as assessed by quantitative RT-PCR and western blotting, respectively. DAXX protein levels did not change appreciably with differentiation (Figure 2d). Thus, myogenic differentiation is associated with repression of PML expression, loss of PML NBs and redistribution of PML NB components such as DAXX.

To determine whether PML loss was sufficient for DAXX association with chromocentres in undifferentiated C2C12 myoblasts, we silenced PML with shRNA and observed DAXX localization (Figure 2e). In cells expressing a control hairpin, DAXX and PML colocalized as expected at PML NBs (Figure 2e, arrowheads). In shPML C2C12 cells, PML NBs were not observed and DAXX was found associated with condensed chromatin (Figure 2e, arrows), consistent with previous reports.^2, 30^ Thus, PML loss in myoblasts is sufficient for DAXX relocalization during myogenesis.

**Figure 3:**
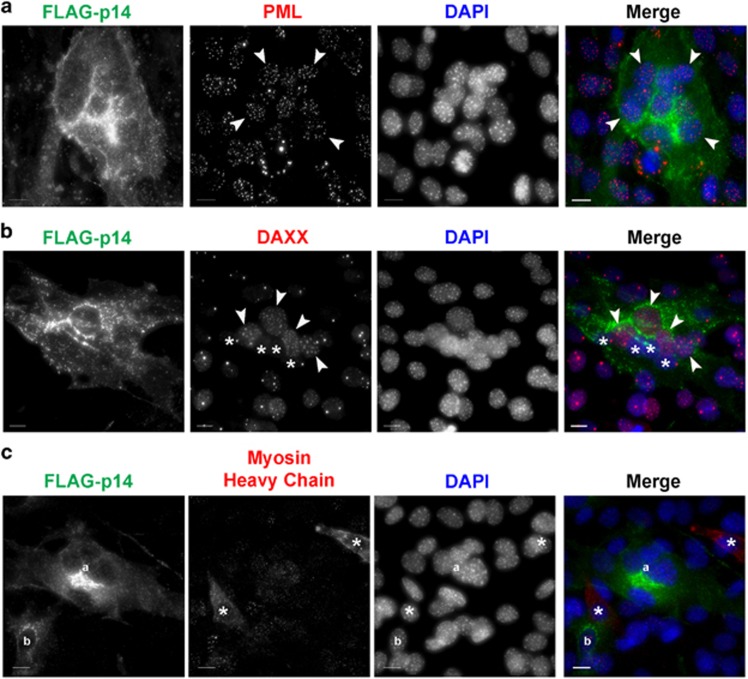
Fusion is not sufficient for PML loss. C2C12 cells were transfected with a plasmid encoding the fusogenic FLAG-tagged RRV-p14 protein and prepared at 36 h post transfection for immunofluorescence microscopy for RRV-p14 (anti-FLAG), PML, DAXX or myosin heavy chain. DNA was visualized with DAPI. Scale bar =10 *μ*m. (**a**) Examples of PML expression within syncytial nuclei is indicated with arrowheads. (**b**) Examples of syncytial nuclei with DAXX relocalization (arrowheads) or DAXX loss (asterisks) are indicate. (**c**) RRV-p14-transfected cells were assessed for expression of the differentiation marker myosin heavy chain. Syncytial (**a**) and individual (**b**) RRV-p14-positive cells did not express myosin heavy chain. Asterisks indicate untransfected cells that express myosin heavy chain

We next examined if PML loss was associated with the differentiation program or a consequence of the cell–cell fusion event. First, we induced cell–cell fusion through ectopic expression via transfection of the reptilian reovirus (RRV) fusion-associated small transmembrane (FAST) protein p14, a promiscuous fusogen that causes efficient cell to cell fusion and syncytium formation in cells of many types.^33, 34^ In C2C12 cells, expression of RRV-p14 resulted in multinucleated syncytium formation within 36 h (Figure 3a). RRV-p14-induced fusion led to oval shaped syncytia with centrally clustered nuclei that differed from linear arrangement of nuclei in normal myotubes, demonstrating that the differentiation program also helps control the architecture of the myotubes post fusion. Importantly, within RRV-p14-mediated syncytia we did not observe a loss of PML NBs, indicating that fusion of myoblasts is not sufficient to drive PML loss (Figure 3a). DAXX localization in RRV-p14-mediated syncytial cells was heterogeneous, ranging from no apparent change to redistribution and loss (Figure 3b). In addition, RRV-p14 did not induce the differentiation program as assessed by myosin heavy chain expression (Figure 3c), indicating that the observed DAXX relocalization and loss resulting from RRV-p14-mediated cell–cell fusion was not dependent on differentiation. Thus, forced myoblast fusion results in only partial relocalization of DAXX and has no effect on PML loss, suggesting other developmentally programmed changes are required for PML NB loss and full redistribution of DAXX during myogenesis.

**Figure 4:**
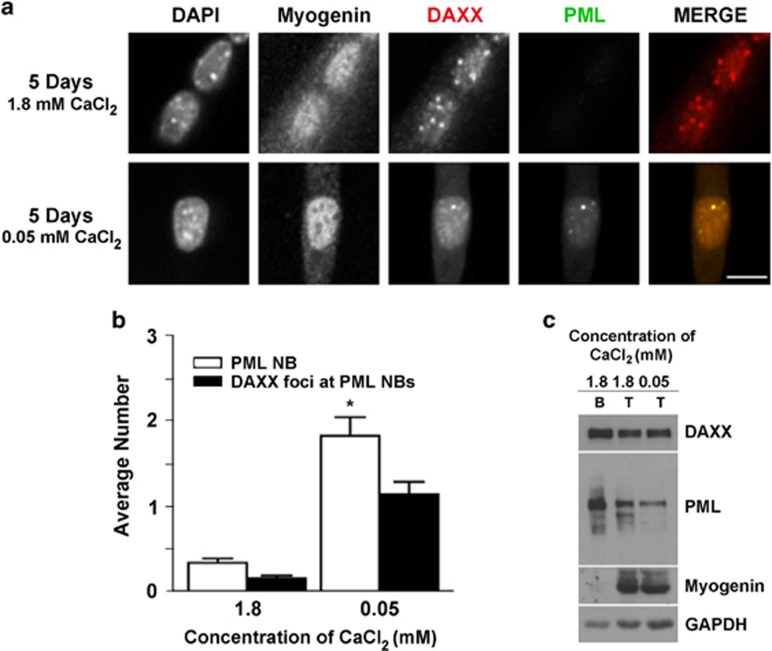
DAXX is retained at PML NBs under low-calcium conditions. (**a**) Immunofluorescence microscopy of C2C12 cells differentiated for 5 days under control (1.8 mM) or myotube-inhibiting low-calcium (0.05 mM) conditions. Under low-calcium conditions, PML NBs (green) were increased relative to control cells and a fraction of DAXX (red) was retained there. Scale bar=10 *μ*m. (**b**) Quantification of the number of PML NBs and DAXX foci at PML NBs. Data are presented as the mean±standard error, *n*=3, *P<0.01. A minimum of 100 cells were analyzed for each treatment. (**c**) Western blot analysis of DAXX and PML protein levels following differentiation under the two conditions. B, myoblasts; T, myotubes

Calcium is a key regulator of myogenesis and reduced extracellular calcium can inhibit differentiation and interfere with myoblast fusion.^35, 36, 37^ To examine the effects of calcium in our system, C2C12 myoblasts were differentiated for 5 days in media with a normal calcium concentration (i.e., 1.8 mM) that promotes fusion or in low-calcium media (0.05 mM) that would allow activation of the differentiation program (i.e., myogenin expression) but prevent cell–cell fusion. Cells grown in control DM (1.8 mM Ca) showed the expected reduction in the number of PML NBs and relocalization of DAXX to chromocentres in the fully differentiated, multinucleated myotubes (Figure 4a). Inhibition of fusion by low-calcium treatment did not prevent dramatic PML loss in most cells (Figure 4a); however, some unfused myogenin-positive cells exhibited PML NBs that were associated with DAXX and therefore the number of PML NBs was significantly higher in C2C12 cells differentiated under low-calcium conditions (Figure 4b; *P*<0.001). Western blot analysis of whole cell lysates from myoblasts, control, and experimental myotubes showed similar PML protein expression levels in the myotube samples, and a decrease in PML levels relative to myoblasts (Figure 4c). In contrast, the expression level of DAXX remained unchanged under low-calcium conditions.

**Figure 5:**
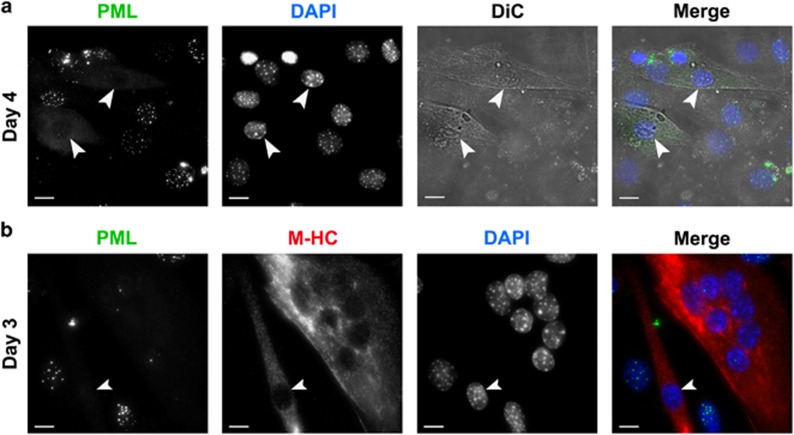
PML loss precedes myotube fusion. (**a**) Immunofluorescence and differential interference contrast (DiC) microscopy of C2C12 cells differentiated for 4 days. Arrows indicate individual unfused cells with loss of PML NBs (green). (**b**) Immunofluorescence microscopy of C2C12 cells differentiated for 3 days and immunostained for PML and myosin heavy chain (M-HC) as indicated. Arrow indicates an unfused but differentiated cell with PML NB loss. DNA was visualized with DAPI. Scale bars=10 *μ*m

The results with low-calcium and RRV-p14 indicated that PML loss likely occurs as part of the differentiation program. To further explore this hypothesis, we first performed a closer examination of differentiated C2C12 cells after 4 days of horse serum treatment and observed unfused individual cells that contained no PML NBs (Figure 5a). These unfused, PML negative cells were more apparent at 3 days post differentiation and were associated with the expression of the late differentiation marker myosin heavy chain (Figure 5b). Together, these results indicate that the loss of PML protein, and by extension PML NBs, occurs as a consequence of the muscle differentiation program.

**Figure 6:**
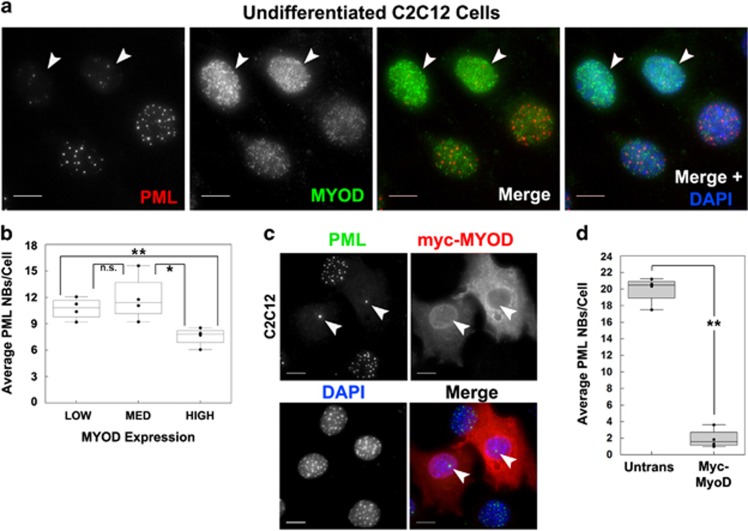
MyoD expression is sufficient for PML NB loss. (**a**) Undifferentiated C2C12 cells were immunostained for PML and MyoD as indicated. Arrows indicate cells with higher MyoD expression and lower PML NBs. (**b**) Quantification of the average number of PML NBs in C2C12 cells based on high (HI), medium (MED), or low (LOW) MyoD expression. *n*=4, **P<0.01, *P<0.05, n.s.=not significant. (**c**) C2C12 cells were transfected with plasmid expressing myc-tagged MyoD and prepared for immunofluorescence microscopy 48 h post transfection. DNA was visualized with DAPI. Scale bars=10 *μ*m. (**d**) Quantification of the average number of PML NBs in C2C12 cells±expression of myc-tagged MyoD or transfection control plasmid (GFP). *n*=4, **P<0.01

We next examined if PML NBs loss was associated with earlier differentiation markers such as MyoD, a master regulator of myogenesis.^38^ Consistent with the hypothesis that loss of PML is a regulated event during the muscle differentiation program, C2C12 cells grown in growth media (GM) showed variable levels of MyoD, and those expressing more MyoD had reduced numbers of PML NBs (Figure 6a, arrows). We quantified the average number of PML NBs in C2C12 cells with low, medium or high MyoD expression and found that high MyoD expression (>1 S.D. from the mean) was associated with a significant decrease in PML NB number (avg 7.6) when compared to medium (avg 11.9, *P*=0.040) or low (avg 10.7, *P*=0.008) MyoD-expressing cells (Figure 6b). To determine whether MyoD expression was sufficient to cause PML NB loss, we expressed myc-tagged murine MyoD in C2C12 cells and observed significant PML NB loss (Figure 6c), with myc-MyoD-transfected cells having 85% fewer PML NBs than GFP-transfected cells (Figure 6d).

**Figure 7:**
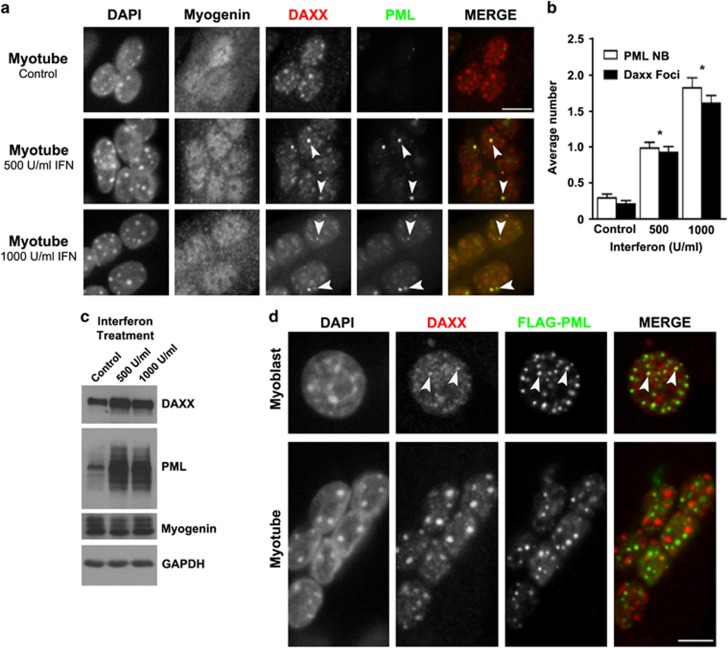
DAXX can associate with PML NBs in myotubes in the presence of interferon, but not ectopically expressed PML cDNA. (**a**) Fully differentiated C2C12 myotubes were treated with the indicated concentration of interferon alpha for 24 h and then prepared for immunofluorescence microscopy. Arrowheads indicate association of DAXX with reformed PML NBs in interferon-treated cells. Scale bar=10 *μ*m. (**b**) Quantification of the number of PML NBs and DAXX foci at PML NBs in C2C12 myotubes the presence or absence of interferon. Data are presented as the mean±standard error, *n*=3, *P<0.01. A minimum of 120 cells were analyzed for each treatment. (**c**) Western blot analysis of DAXX and PML protein levels in differentiated C2C12 cells treated with the indicated concentration of interferon. (**d**) Immunofluoresence microscopy of C2C12 myoblasts transfected with FLAG-PML and differentiated into myotubes according to standard conditions. In myoblasts, DAXX (red) is enriched in ectopic PML (FLAG, green) structures (arrowheads). The presence of PML-containing structures in differentiated myotubes failed to recruit DAXX. Under these conditions, DAXX remained at chromocentres. Scale bar=10 *μ*m

To further explore the link between PML NB loss and DAXX relocalization during myogenesis, we attempted to increase PML expression by treating myotubes with interferon, which can induce PML expression and increased both the size and number of PML NBs.^39^ The rationale being that induction of PML expression in this manner might inhibit the relocalization of DAXX to chromocentres by enabling PML NBs to form and sequester DAXX. To this end, we treated day 5 myotubes with 500 or 1000 IU/ml of interferon alpha for 24 h and analyzed PML and DAXX expression and localization (Figure 7a). Interferon treatment partially restored PML NBs in myotube nuclei in a dose-dependent manner, resulting in the formation of only 1–2 bodies per nucleus (Figure 7b). The reestablishment of these PML NBs was sufficient to support partial relocalization of DAXX to the reformed PML NBs (Figure 7a, arrowheads). Consistent with previous studies^40, 41, 42^ interferon induced the expression of PML and, to a lesser degree, DAXX (Figure 7c). Given the marked increase in PML protein, but only modest reestablishment of PML NBs, it is likely that additional mechanism(s) may also restrict PML NB formation in myotubes.

As we were unable to significantly increase the number of PML NBs in myotubes with IFN treatment, we next sought to determine whether forced PML expression could alter DAXX relocalization and/or myoblast differentiation. To this end, we transfected C2C12 myoblasts with plasmids expressing FLAG-tagged murine PML and allowed the cells to differentiate for 5 days. Ectopic expression of PML in myoblasts caused an increase in PML NB size and number but did not affect DAXX association with PML NBs (Figure 7d). Ectopic expression of PML also did not prevent myotube formation, indicating that PML loss is not required for differentiation. Expression of FLAG-PML in myotubes was associated with an increase in PML NB number; however, the total number of PML NBs in transfected syncytial nuclei was greatly reduced compared with transfected myoblasts. In PML-transfected myotubes, however DAXX was not significantly associated with PML NBs. Together, these data lead us to propose that differentiation-specific alterations to PML NBs, DAXX and/or chromocentres actively promote the displacement of DAXX from PML NBs and its association with chromocentres.

**Figure 8:**
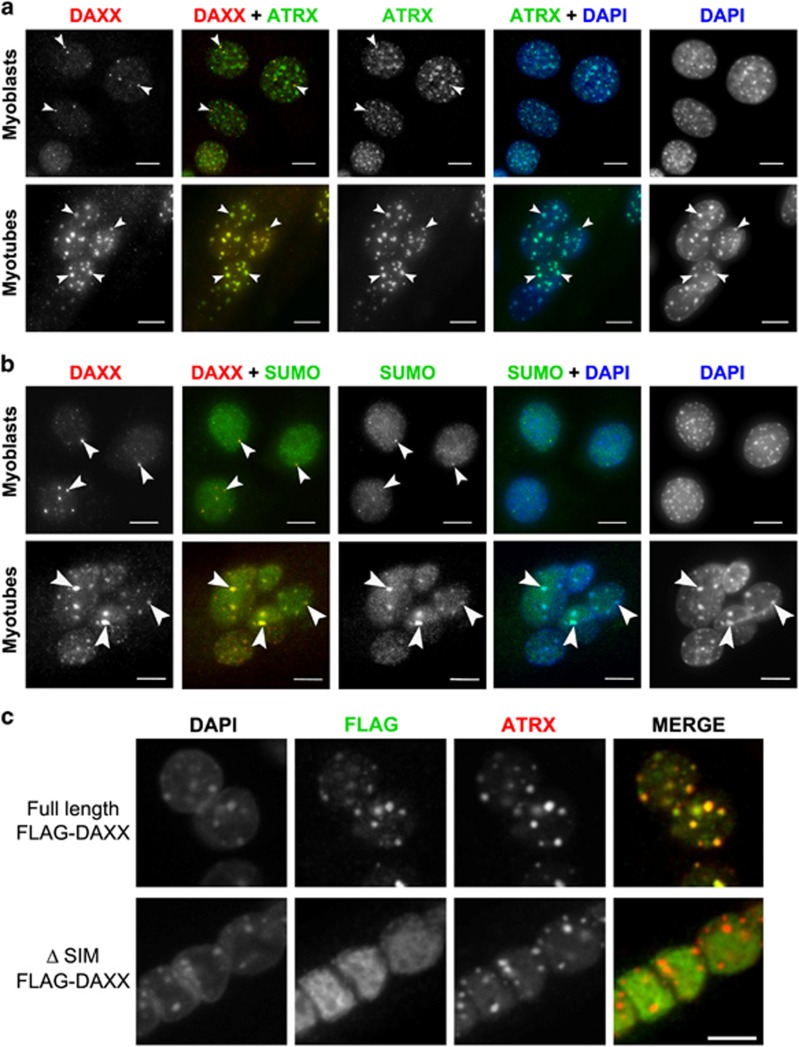
DAXX associates with ATRX and SUMO at heterochromatin in myotubes and this interaction requires the DAXX SUMO-interacting motif (SIM). (**a**) Immunofluoresence microscopy of ATRX and DAXX localization in C2C12 myoblasts and myotubes. Arrowheads indicate colocalization between ATRX and DAXX. (**b**) Imunofluoresence microscopy of SUMO and DAXX localization in C2C12 myoblasts and myotubes. Arrowheads indicate colocalization between SUMO and DAXX. (**c**) C2C12 myoblasts were transfected with FLAG-tagged full-length DAXX or DAXX ΔSIM expression plasmids prior to differentiation into myotubes. Scale bars=10 *μ*m

DAXX forms a complex with *α*-thalassemia and mental retardation X-linked (ATRX) DNA helicase to act as a histone chaperone for the histone variant H3.3 to facilitate its deposition into pericentric heterochromatin, telomeres, silenced imprinted loci and endogenous retrovirus elements.^43, 44, 45, 46^ Like DAXX, ATRX can associate with both heterochromatin and PML NBs.^30, 47^ We therefore wanted to investigate if PML NB loss during differentiation disrupted the association of ATRX and DAXX at heterochromatin. In myoblasts, we found that ATRX is localized to heterochromatin, as well as associated with DAXX at PML NBs (Figure 8a). However, in myotubes that lack PML NBs, ATRX is associated with DAXX at chromocentres, consistent with the histone chaperone and recruitment function of the ATRX-DAXX complex. As DAXX is recruited to PML NBs through binding SUMOylated PML^2, 3^ we also evaluated the localization of SUMO1 and DAXX in C2C12 myoblasts and myotubes (Figure 8b). SUMO was associated with DAXX foci in both myoblasts and myotubes. These results are consistent with DAXX being SUMO modified^48^ and/or associating with SUMOylated target proteins via the DAXX SIM.^3, 49^ To help distinguish between these two possibilities, we also expressed FLAG-tagged full-length DAXX and a SIM-deleted mutant (DAXX-ΔSIM) in C2C12 cells and differentiated them for 5 days (Figure 8c). Similar to endogenous DAXX, FLAG-DAXX was enriched in ATRX-containing chromocentres; whereas, the DAXX-ΔSIM mutant exhibited a nuclear diffuse localization and was not enriched in chromocentres. Therefore, the redistribution of DAXX to chromocentres during skeletal muscle differentiation occurs concomitantly with loss of PML and requires the C-terminal SIM domain of DAXX.

## Discussion

Despite evidence that PML has a role in developmental processes, the fate of PML during muscle differentiation has not been documented in detail. In this study, we examined the fate of PML and PML NBs during skeletal muscle differentiation using the murine C2C12 myoblast cell line as our model system and found that there is a profound loss of PML NBs during myogenesis. Here, we demonstrated that the loss of PML NBs is associated with expression of the differentiation markers MyoD and myogenin in both myoblasts and myotubes and that ectopic expression of MyoD is sufficient to cause PML NB loss. Furthermore, induction of cell–cell fusion via the reovirus p14 FAST protein, in the absence of muscle differentiation, failed to cause PML loss. Thus, PML NB loss is not a consequence of cell fusion during muscle differentiation but likely a programmed event triggered by MyoD. We also observed a marked relocalization of DAXX to HP1*α*-containing chromocentres that coincided with loss of PML NBs in myotubes. These programmed changes in PML NBs that we observed are likely conserved among mammals, as a study by Homma et al.^50^ published during revision of this manuscript described a similar reduction in PML NB number during human primary myoblast differentiation. Thus, we propose a model in which activation of the myogenic differentiation program by MyoD in myoblasts results in the loss of PML NBs in myotubes and the concomitant redistribution of the PML NB component protein DAXX to heterochromatin domains.

During differentiation, PML NB loss is associated with loss of PML mRNA and protein expression (Figure 2). Although these data support a model for PML NB loss through reduced *Pml* gene expression, increasing PML expression with interferon (Figures 7a and b) and plasmid-expressed PML (Figure 7d) was only able to partially restore PML NB formation relative to unfused myoblasts. Thus, in addition to transcriptional repression of PML in myotubes, other mechanisms (e.g., protein modification) may prevent normal PML NB formation even in the presence of abundant PML protein.

The striking loss of PML NBs during muscle differentiation and the link to MyoD expression leads us to speculate that PML loss may have a role in regulating the muscle-specific gene expression profile. Indeed, activation of the muscle differentiation program, triggered in part by MyoD, results in the differential expression of hundreds of genes, withdrawal of differentiated cells from the cell cycle and a reorganization of heterochromatin domains. PML NBs have been shown to coordinate transcriptional activity, regulate cell cycle and senescence programs and have roles in sequestering and/or the post-translational modification of chromatin modifying enzymes at PML NBs such as DAXX, ATRX, and the HDACs. Therefore, loss of PML NBs during muscle differentiation might be necessary to properly execute any or all of the above cellular changes. Although beyond the scope of the present work, future studies could directly investigate the possibility that downregulation of PML and concomitant loss of PML NBs has a role in altering the transcriptome during myogenesis.

One consequence of PML NB loss during skeletal muscle differentiation uncovered in this study is the relocalization of DAXX from PML NBs to chromocentres. DAXX localization to PML NBs can regulate DAXX functions by enhancing its pro-apoptotic activity^51, 52^ and inhibiting its repressor activity.^53^ In the absence of PML, DAXX is found at chromocentres where its repressor function is restored,^53^ and recent reports implicate PML in the regulation of the histone chaperone activity of the ATRX-DAXX complex.^54, 55^ Consistent with reports in other experimental cell systems,^2, 30^ our data indicate that loss of PML in C2C12 myoblasts is sufficient to cause DAXX relocalization to chromocentres; however, our data also show that DAXX association with chromocentres occurs in reovirus p14-induced C2C12 syncytial cells in the presence of PML NBs. Therefore, the accumulation of DAXX at chromocentres in myotubes may be initiated following syncytia formation as a programmed developmentally process that is completed and/or reinforced by PML NB loss.

We also demonstrate that reintroduction of PML via IFN treatment or ectopic expression in differentiated myotubes is able to only partially restore DAXX localization to PML NBs and cannot fully prevent/reverse DAXX association with heterochromatin (Figure 7). This might indicate that post-translational modification of DAXX and/or the inability to re-establish a ‘threshold' number of PML NBs in these experiments impairs DAXX association with PML in IFN-treated or PML-transfected myotubes. Regardless of the mechanism of DAXX association with chromocentres during differentiation, it is possible that DAXX relocalization contributes to the global chromatin changes that accompany differentiation. It has been shown that DAXX can reside at pericentric heterochromatin and, through its association with ATRX, is responsible for the deposition of H3.3 into chromatin containing major satellite repeats.^2, 45^ In cycling cells, the relocalization of DAXX from PML NBs to heterchromatin domains occurs in late S-phase and requires the C-terminal SIM domain of DAXX.^30^ We found that DAXX localization to heterochromatin domains enriched in ATRX also occurred in post-mitotic myotubes concomitant with PML loss and similarly required the SIM domain of DAXX (Figure 8). Although the functional significance of the relocalization of DAXX to chromocentres in the context of muscle differentiation remains to be determined, DAXX is known to be required to maintain the structural integrity of constitutive heterochromatin domains.^56^ Recently, the chromodomain helicase DNA-binding domain 2 chromatin remodeling enzyme was identified as a myogenic cell fate determinant by specifically mediating the incorporation of H3.3 into the promoters of myogenic genes.^57^ Interestingly, the depletion of H3.3 impaired myotube formation. Furthermore, DAXX can inhibit myogenic differentiation through interactions with the E2A pro-myogenic transcription factor and recruitment of histone deacetylases to E2A-dependent promoters.^58^ Therefore, we speculate that DAXX may be recruited to chromocentres during muscle differentiation to mediate the large-scale heterochromatin reorganization events that mark myogenesis, which may explain why the loss of DAXX has such devastating consequences in development.^59^ Similarly, mice with skeletal muscle-specific conditional ATRX knock-out show myogenic defects,^60^ further implicating DAXX and ATRX in regulation of normal muscle development.

Although our study is among the first descriptions of the developmentally controlled loss of PML during muscle differentiation there is some evidence that PML loss occurs during neuronal development as well. In tissue culture models^61^ and in the developing mouse brain,^29^ PML expression is restricted to the neural progenitor cells and is absent in mature neurons. Together, these data support a model in which developmentally regulated PML NB loss may be a common feature of terminally differentiated cells. Further, high-grade tumors of the breast, prostate, and lung are often associated with lower PML expression.^62^ Thus, a better understanding of how PML loss affects the regulatory program during myogenesis may provide a paradigm for understanding the consequence of PML loss in other contexts, such as during the loss of PML protein expression in high-grade malignancies.

## Materials and Methods

### Cell culture

C2C12 myoblasts (ATCC cat# CRL-1772) were cultured in GM: DMEM (Gibco, Burlington, ON) supplemented with 10% FBS (Wisent, St-Bruno, QC), 10 *μ*g/ml penicillin and streptomycin (Wisent), and 2 mM l-glutamine (Wisent). To induce myogenesis, and generate fully differentiated myotubes, myoblasts were grown to confluence and cultured in low-mitogen DM: DMEM (Gibco) containing 2% horse serum (Wisent), 10 *μ*g/ml penicillin and streptomycin (Wisent), and 2 mM l-glutamine (Wisent). Cells were differentiated for 3–5 days before analysis. In all differentiation experiments, media was replaced every 48 h. Cells were grown on glass coverslips for immunofluorescence microscopy, electron microscopy, and transfections. For transfections, myoblasts were seeded to 50% confluency on glass coverslips and transiently transected (Lipofectamine 2000 Transfection Reagent, Invitrogen, Burlington, ON, Canada) with the specified purified (Qiagen Plasmid Midi Kit, Qiagen, Germantown, MD, USA) FLAG plasmids. For interferon treatment, myoblasts were first differentiated for 5 days in DM. Interferon-*α* was added to fresh DM to a final concentration of either 500 or 1000 U/ml. Myotubes were treated with interferon for 24 h prior to preparation for immunofluorescence and western blot analysis. To prepare the low-calcium GM and DM, calcium-free DMEM (Wisent) was used and supplemented as described above. Low-calcium GM and DM was achieved by supplementation with CaCl_2_ to a final concentration of 0.05 mM. Prior to immunofluorescence and western blot analysis, cells were cultured as described above. U2OS cells were acquired from ATCC (cat# HTB-96) and maintained in DMEM supplemented with 10% FBS.

### Quantification of PML NBs and DAXX foci

Quantification of PML NBs and DAXX foci in low-calcium and interferon-treated cells was determined by determining the average number of each respective structure in at least 30 nuclei from three independent experiments. The values are presented as the mean±standard error.

### Quantification of MyoD and PML levels

Undifferentiated C2C12 cells were grown on coverslips in GM prior to fixation and preparation for immunofluorescence microscopy. Images of random fields of view were analyzed with FIJI software.^63^ In brief, nuclei were identified as regions of interest (ROI) based on the DAPI staining signal. At least 100 individual nuclei wholly contained within a field of view were analyzed for each of four biological replicates. Within each defined nuclear ROI the average intensity of the MyoD signal was calculated and the number of PML NBs was determined. These paired values for each nuclei were sorted by MyoD intensity and binned based on values less than one standard deviation (1 S.D.) from the mean (low), within 1 S.D. (medium) and >1 SD. (high) and the average number of PML NBs for high, medium and low MyoD-expressing cells was calculated.

### Transfection and plasmid constructs

Full-length CMV-myc-tagged murine *Myod1* was a gift from Andrew Lassar (Addgene plasmid #8399). Full-length mouse PML isoform 1 cDNA (Mammalian Gene Collection, MCG 13700, GenBank: BC020990.1) was obtained from The Centre for Applied Genomics (TCAG, The Hospital for Sick Children) and subcloned into the pcDNA3.1(+)-FLAG expression construct (Life Technologies Inc., Burlington, ON, Canada). C-terminal FLAG-tagged RRV-p14 was expressed from a pcDNA3.1 plasmid as previously described.^64^ To clone full-length mouse DAXX, total RNA was isolated from wild type MEFs using TRIzol Reagent (Life Technologies Inc.) and first-strand cDNA was generated using a long-range cDNA synthesis kit (Qiagen). Full-length DAXX, and the DAXX-ΔSIM mutant were subcloned into the pcDNA3.1(+)-FLAG expression construct (Life Technologies Inc.). All cloned plasmids were confirmed by sequencing. Transfected cells were prepared for immunofluorescence microscopy at 24–48 h post transfection. Control (cat# RHS4346) and PML-targeting (Clone ID: V2LMM-59460) shRNA-encoding plasmids were acquired from Open Biosystems (Dharmacon) in pGIPZ expression vectors and expressed in C2C12 cells by transfection. All cloned plasmids were confirmed by sequencing. For transfections, myoblasts were seeded to 50% confluency on glass coverslips and transiently transfected (Lipofectamine 2000 Transfection Reagent, Invitrogen) with the specified purified (Qiagen Plasmid Midi Kit, Qiagen) FLAG plasmids.

### Immunofluorescence microscopy

Cells were fixed in 2% paraformaldehyde (Electron Microscopy Sciences, Hatfield, PA, USA) in PBS (Wisent) for 10 min at room temperature (RT) and permeabilized in 0.5% Triton X-100 (BioShop, Burlington, ON, Canada) in PBS for 5 min at RT. Primary antibodies used were rabbit anti-DAXX (M-112; cat# sc-7152, Santa Cruz Biotechnology, Dallas, TX, USA, 1:200), mouse anti-PML (clone 36.1-104; cat# mab3738, EMD Milipore, 1:200), rabbit anti-myosin heavy chain (ab91506, Abcam, Eugene, OR, USA, 1:200), goat anti-myogenin (Santa Cruz Biotechnology, 1:200), mouse anti-myc (clone 9E10, Santa Cruz Biotechnology, sc-40, 1:200), rabbit anti-MyoD (Santa Cruz Biotechnology, sc-760, 1:200), mouse anti-ATRX (clone D-5, Santa Cruz Biotechnology, sc-55584, 1:200), mouse anti-SUMO-1 (clone 21C7, Zymed, San Francisco, CA, USA, #33-2400 1:200), and mouse anti-FLAG (M2; cat# F1804, Sigma-Aldrich, 1:1000). Secondary antibodies used were rabbit, sheep, or mouse Cy2, Cy3, and Cy5 (Jackson Laboratories, West Grove, PA, USA, 1:800). Cells were incubated in 50 *μ*g/ml DAPI and mounted using anti-fade reagent (buffered glycerol with 4% *n*-propyl gallate). Images were collected on either an Olympus IX81 inverted microscope equipped with a Cascade II CCD camera (Photometrics, Tucson, AZ, USA) using either a 60 × or 100 × oil-immersion objective lenses, or Zeiss Cell Observer inverted microscope equipped with a CoolSNAP HQ2 CCD camera (Photometrics) using a 63 × oil-immersion lens. For image capture, either MetaMorph Microscopy Automation & Image Analysis Software (Molecular Devices, Palo Alto, CA, USA) or Slidebook 5.1 (Intelligent Imaging Innovations, Boulder, CO, USA) was used to collect images. Images were then processed with Volocity 3D Image Analysis Software (PerkinElmer, Boston, MA, USA) and Photoshop (Adobe, San Jose, CA, USA). Graphs and statistics were constructed using GraphPad Prism (GraphPad Software Inc, La Jolla, CA, USA) and Excel (Microsoft, Seattle, WA, USA). Quantification statistics are reported as S.E.M. and *P-*values were generated from student's *t*-tests.

### Quantitative PCR

Control (day 0) and differentiated (day 5) C2C12 were grown in 10 cm dishes and were immediately solubilized in TRIzol reagent (Life Technologies Inc., cat#15596-026). Total RNA was extracted using the PureLink RNA Mini Kit (Life Technologies Inc., cat#1218025) following the manufacturer's instructions. RNA yield and quality were assessed by determining the 260 nm/280 nm and 260 nm/230 nm absorbance ratios. Further qualitative evaluation of RNA purity was conducted using an ethidium bromide-stained 1.5% agarose gel to visualize 28S and 18S ribosomal RNA bands. cDNA was generated from 1 *μ*g RNA using the iScript Reverse Transcription Supermix protocol (Bio-Rad Laboratories Ltd., Mississauga, ON, Canada, cat#170-8841). Quantitative real-time PCR for *Pml*, *Myog*, *Pax7,* and reference genes *Hprt*, *Rps12*, and *Tbp* was conducted using SsoAdvanced Universal SYBR Green Supermix (Bio-Rad Laboratories Ltd., cat#172-5271) on a CFX96 Touch Real-Time PCR Detection System (Bio-Rad Laboratories Ltd.). Reference gene selection (*Hprt*, *Rps12*, and *Tbp*, Mean CV=0.1403, Mean *M*-value=0.3386), primer optimization, and experimental data collection were conducted in triplicate reactions for biological triplicates. Quantitative data analysis was performed on CFX Manager Software (Bio-Rad Laboratories Ltd., v3.1). Primers were designed using Primer 3-Blast (Primer-Blast, National Center for Biotechnology Information, Bethesda, MD, USA) and the following sequences (5'–3') were used: *Pml* (fwd: gacaatgaaacccagaaaattagc, rev: agggagacagctttggagtag), *Myog* (fwd: caggagatcatttgctcg, rev: gggcatggtttcgtctgg), *Pax7* (fwd: tgagttcgattagccgagtgc, rev: tccagacggttccctttgtc), *Hprt* (fwd: atggactgattatggacaggactg, rev: tccagcaggtcagcaaagaac), *Rps12* (fwd: aaggcatagctgctggaggtgtaa, rev: agttggatgcgagcacacacagat), *Tbp* (fwd: tgcacaggagccaagagtgaa, rev: cacatcacagctccccacca)

### Western blotting

Cells grown in 10-cm culture dishes were harvested and solubilized with 9 M Urea in 10 mM Tris-Cl, pH 6.8. Samples were quantified using the Bio-Rad Protein Concentration Assay (Bio-Rad Laboratories) and diluted to 10 *μ*g/20 *μ*l in 9 M Urea containing a 6 × SDS-PAGE loading buffer. Protein samples were resolved on 10% SDS-PAGE gels and transferred onto nitrocellulose membranes (GE Healthcare, Mississauga, ON, Canada). Membranes were blocked overnight in 5% skim milk powder in TBST (0.05% Tween-20, Bio-Rad). Primary antibodies used were rabbit anti-DAXX (Santa Cruz Biotechnology, Dallas, TX, USA), mouse anti-PML (EMD Milipore, Etobicoke, ON, Canada), goat anti-myogenin (Santa Cruz Biotechnology), and GAPDH (Sigma). Secondary antibodies used were mouse and rabbit anti-HRP (Sigma). Detection was performed using the Western Lightning Plus ECL system (PerkinElmer, Woodbridge, ON, Canada).
